# *Bothrops jararacussu* Venom Inactivated by High Hydrostatic Pressure Enhances the Immunogenicity Response in Horses and Triggers Unexpected Cross-Reactivity with Other Snake Venoms

**DOI:** 10.3390/toxins17020088

**Published:** 2025-02-13

**Authors:** Ricardo Teixeira-Araujo, Marisa Carvalho Suarez, Carlos Correa-Netto, Luis Eduardo Ribeiro da Cunha, Debora Foguel, Russolina Benedeta Zingali

**Affiliations:** 1 Instituto de Bioquímica Médica Leopoldo de Meis (IBqM) Universidade Federal do Rio de Janeiro, Rio de Janeiro 21941-599, Brazil; rcdo.araujo@gmail.com (R.T.-A.); netto@bioqmed.ufrj.br (C.C.-N.); foguel@bioqmed.ufrj.br (D.F.); 2 Instituto Nacional de Ciência e Tecnologia de Biologia Estrutural e Bioimagem (Inbeb), Universidade Federal do Rio de Janeiro, Rio de Janeiro 21941-599, Brazil; 3 Instituto Vital Brazil, Niterói 24230-410, Brazil; lcunha@nthink.com.br; 4 Campus Duque de Caxias Professor Geraldo Cidade, Universidade Federal do Rio de Janeiro, Duque de Caxias 25240-005, Brazil; mcarvalhosuarez@yahoo.com.br

**Keywords:** toxicity inactivation, immunization, antivenom, neutralization, Najussu, Prejussu

## Abstract

High hydrostatic pressure (HHP) has been used for viral inactivation to facilitate vaccine development when immunogenicity is maintained or even increased. In this work, we used HHP to inactivate *Bothrops jararacussu* venom. Our protocol promotes the loss of or decrease in many biological activities in venom. Horses were immunized with pressurized venom, and in contrast to native venom, this procedure does not induce any damage to animals. Furthermore, the serum obtained with pressurized venom efficiently neutralized all biological activities of *B. jararacussu* venom. Antibody titrations were higher in serum produced with pressurized venom compared to that produced by native venom, and this antivenom was not only effective against the venom of *B. jararacussu* but against the venom of other species and genera. In conclusion, our data show a new technique for producing hyperimmune serum using venom inactivated by HHP, and this method is associated with a reduction in toxic effects in immunized animals and higher potency.

## 1. Introduction

Antivenom is the only recognized and specific snakebite treatment [1]. There is no doubt that the scarcity of effective snake antivenoms that are regionally appropriate for use remains a public health problem worldwide [2,3], especially in African and Asian countries, where snakebites still cause death or morbidity, and these issues are neglected by governments and antivenom producers [4,5,6]. Since 2012, the WHO has provided publications on this matter. The WHO Expert Committee on Biological Standardization (ECBS) recognized that, to manage this crisis better, some challenges would need to be correctly overcome to ensure the quality, safety, and efficacy of the antivenoms [7]. In this regard, the production of inexpensive and specific antivenoms is desired.

One aspect that is relevant and little-discussed in the scientific community is the effective cost of using large animals to produce antivenoms [7]. Equines are the preferred animals for obtaining hyperimmune plasma; their large stature makes it possible for them to take a large volume of plasma [7,8]. Therefore, any loss of animals is economically and clinically significant. Although the WHO recommends the necessary provision of individual veterinary clinical care and adequate support to promote well-being [7,8], complications such as the development of ulcers, abscesses, local necrosis, and hemorrhages, which either limit the physical condition of the animal or culminate in its death, are monitored throughout the entire immunization process [8,9,10]. In addition, the animal’s use window is relatively narrow due to the toxicity of the venom [11,12]. As a result, studies have been carried out to obtain antigens with low toxicity and an increased immune response since some snakes have venoms with low immunogenic capacity but high toxicity [13]. The evaluated procedures include iodination and treatment with glutaraldehyde-detoxified scorpion venoms [11,14], the alkylation of histidine residues of *B. jararacussu* venom proteins with p-bromophenacyl bromide [15], irradiation [16], and incubation with formaldehyde [17]. As expected, these studies observed significant toxicity loss and immunogenicity maintenance.

We highlight high hydrostatic pressure (HHP) treatment, which has shown potential in inactivating viruses to develop vaccines. Gaspar et al. (2008) used hydrostatic pressure for viral inactivation and vaccine development. The treatment abolished the infectivity of the yellow fever virus in mice. In addition, these animals exhibited complete protection against the lethal challenge of the virus [18]. Dumard et al. (2013) used the H3N8 avian influenza virus inactivated by HHP to produce the vaccine. The results were impressive since the immunized animals were completely protected from the challenge of infection by the native virus [19]. Later, Dumard et al. (2017) found that both particles have similar structural stability and that HHP promotes structural changes, concluding that HHP can be a valuable tool for vaccine development, as it induces small and reversible structural changes associated with the partial preservation of its biological activities and thereby potentiating their immunogenic response by abolishing their infectiousness [20]. Despite the potential application of HHP for vaccine production and its use in several studies with different types of proteins [21,22,23], only a few articles have described the effect of HHP upon purified toxins from venomous animals, generally in structural studies [24,25]. Ruan et al., 1998, showed that an HHP above 400 MPa applied to a purified venom PLA_2,_ followed by intrinsic fluorescence, revealed that the tryptophan was buried deeper inside the protein than when using a standard denaturation process [25]. Also, while under high pressure, the enzyme lost its catalytic activity, but the activity recovered upon release of the pressure [25]. Iwakaga et al., 1998, showed that ShK was affected by HHP (200 MPa), changing the conformation of the disulfide bridge to a compact conformation [24].

Studies on the envenoming caused by *B. jararacussu* have led to recognition of its seriousness [26]. This snake’s venom is classified as predominantly myotoxic, mainly due to the action of Bothropstoxin-I, a PLA_2_ Lys-49 [27,28]. Queiroz et al. (1984) showed that the venom of *B. jararacussu* has intense myotoxic activity, causing necrosis of the striated muscular fiber and delaying its regeneration [28]. These complications can compromise deeper structures, such as muscles and ligaments; in these cases, amputation of the affected limb is recommended [28]. When inoculated into mice’s muscles, this venom promoted more prominent creatine kinase leakage when compared with different bothropic venoms [29]. Milani et al. (1997) analyzed, for a period longer than 20 years, the treatment of 29 victims of proven bites of *B. jararacussu* and found that even high doses of antibothropic antivenom failed to neutralize the toxicity of this venom [30]. Therefore, Dias da Silva et al. (2010) and Correa-Netto et al. (2010) described *B. jararacussu* venom as having low antigenicity [31,32].

Furthermore, this venom has a lower immune response than other bothropics venoms, and the lethal activity of the venom is inadequately neutralized by monospecific (anti-jararacussu) or commercial antibothropic antivenoms [31]. Additionally, dos Santos et al. (1992) showed that the combination of Crotalus and Bothrops antivenoms was more effective in neutralizing the lethal, myotoxic, and procoagulant activities of *B. jararacussu* venom than antibothropic antivenom alone [33]. Because of that, there is still considerable discussion about the quality and effectiveness of antibothropic serum [31].

This work presents a methodology for inactivating the *B. jararacussu* venom using HHP. The pressurized venom was used to obtain the hyperimmune serum. We analyzed the neutralization of the venom’s biological activities and studied its cross-reactivity toward other venoms.

## 2. Results

### 2.1. Establishing the Ideal Conditions for HHP-Induced Inactivation of the Venom

To establish the conditions for inactivating the *B. jararacussu* venom through HHP, we conducted a series of experiments with varying hydrostatic pressures (from 50 to 600 MPa), temperatures (from −10 to 37 °C), and pressurization times (4 to 24 h). All tested conditions are displayed in Supplemental Table S1.

**Figure 1:**
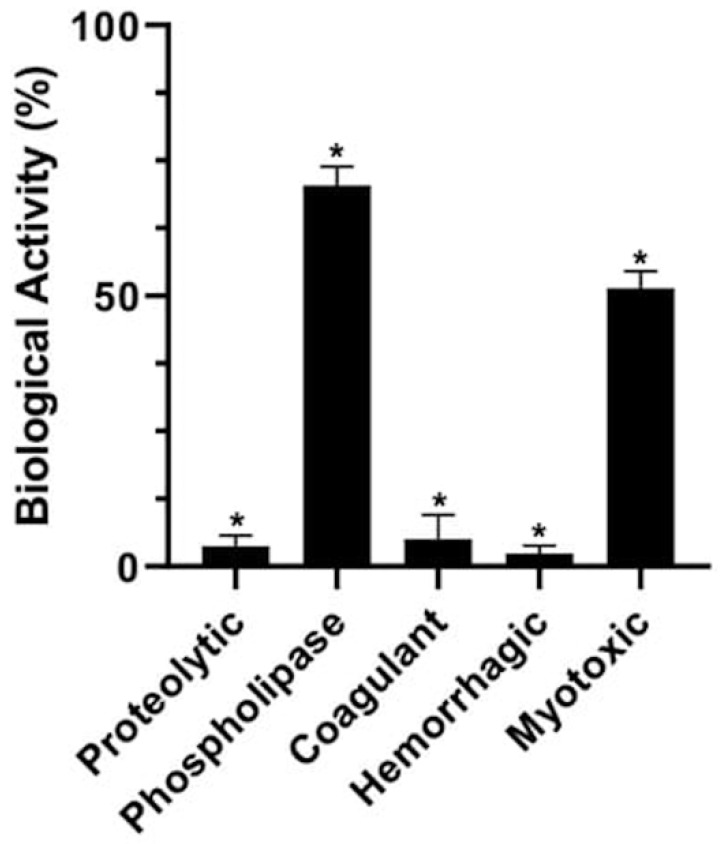
HHP decreases the biological and enzymatic activities of *B. jararacussu* venom. The biological and enzymatic activities displayed in the x-axis were measured in venom samples submitted to 600 MPa/24 h/37 °C (Bj-PV) and compared to those shown by the venom that remained at atmospheric pressure at 37 °C (Bj-NV), presented as a percentage. The data represent the means of three independent experiments. For all activities, (* *p* < 0.05).

After treatments, the enzymatic activities of native and pressurized venoms were evaluated. Venoms exposed to pressures equal to or lower than 290 MPa at all times and all investigated temperatures presented similar phospholipase, proteolytic, and coagulant activities compared to native venom (Table S1). Interestingly, the condition of 600 MPa/24 h/37 °C led to a more significant reduction in the venom’s in vitro and in vivo activities, completely abolishing its proteolytic, clotting, and hemorrhagic effects (Figure 1). Thus, we chose this treatment to continue our evaluation. Phospholipase (Table S1 and Figure 1) and myotoxic activities were only partially inhibited after pressurization (Figure 1). Additionally, the lethal dose that furnishes 50% lethality (DL_50_) was determined for the venom that remained at atmospheric pressure (Bj-NV) and after HHP (Bj-PV); this was, respectively, 87 and 191 µg/20 g body weight, suggesting that after HHP treatment, the venom became half as lethal. Altogether, these data demonstrate that HHP (600 MPa/24 h/37 °C) effectively attenuates the toxic actions of *B. jararacussu* venom, probably due to the decrease in enzymatic/biological activities present in the venom. Thus, the following experiments were performed under this condition.

### 2.2. Structural Characterization of the HHP-Inactivated Venom

#### 2.2.1. Gel Filtration Chromatography Shows Changes in the Profile of Treated Venom

**Figure 2:**
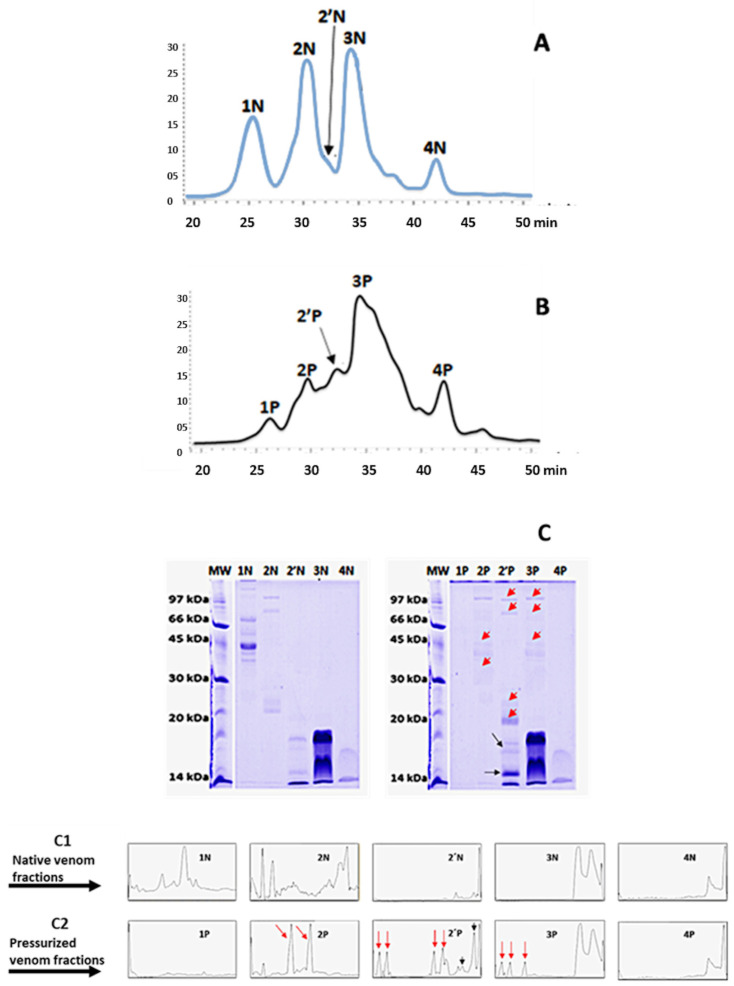
HHP-induced changes in the gel filtration profile and analysis via SDS-PAGE. A total of 6,7 mg of native or pressurized *B. jararacussu* venom was fractionated in a Superose 12 column. In (**A**), native venom of *B. jararacussu* (Bj-NV; atmospheric pressure; 24 h; 37 °C). In (**B**), pressurized venom of *B. jararacussu* (Bj-PV; 600 MPa/24 h/37 °C. Electrophoresis from the chromatographic peaks in (**C**) native venom and pressurized venom. (C1) and (C2) Densitometry of the gels presented in panels C. Red and black arrows show the new bands or increased intensity in the pressurized venom, respectively.

We performed gel filtration chromatography to map the structural changes that HHP promotes in the venom of *B. jararacussu*. The Bj-NV presented four prominent, well-defined peaks, namely, peak 1N, with proteins weighting > 30 kDa; peak 2N and a small shoulder (2N’), with proteins weighting > 20 kDa; and two additional peaks (3N and 4N), with proteins below 20 kDa (Figure 2A). Interestingly, the elution profile of Bj-PV presented significant alterations. While the peaks related to higher-molecular-weight proteins exhibited lower intensities (1P and 2P), the shoulder seen before in Bj-NV increased substantially (peak 2′P). In addition, there was a marked widening of peak 3P (Figure 2B). Only peak 4P was similar to that of native venom, with a slight increase in its intensity. To better visualize all modifications promoted by HHP treatment, an overlay of both profiles is represented in Figure S1.

SDS-PAGE was performed to characterize in more detail the population of proteins present in each elution peak (Figure 2C). As seen, some bands present in peaks 1N (prominent bands at ~40 and 70 kDa) and 2N (prominent bands at ~25, 80, and 100 kDa) of the native venom disappeared after pressurization (1P, 2P). In comparison, proteins below 20 kDa from peaks 3N and 4N remained unchanged after pressure treatment. In contrast, new bands appeared at peaks 2′P and 3P with higher-molecular-weight species (Figure 2). Figure 2C1,C2 shows the densitometry of the gels, emphasizing the changes described.

Mass spectrometry analyses of each peak were performed to understand the changes in the structure induced by HHP in depth (Table S2). High-molecular-weight proteins (30 to 150 kDa), such as metalloproteases (SVMPs), L-amino oxidases (LAAOs), and serine proteinases (SVSPs) were identified in peaks 1N and 2N of Bj-NV. They were also found in peaks 2′P and 3P (Table S3) of Bj-PV, peaks corresponding to proteins with low molecular weights, suggesting that HHP treatment might dissociate oligomeric proteins irreversibly. For instance, metalloproteases are divided into four classes (PI to PIII) according to their domains’ organization and molecular mass. The PI class includes small SVMPs (20–30 kDa), representing only the metalloprotease domain. The PII class (30–50 kDa) has a disintegrin domain in the C-terminal portion. The PIII class (50–90 kDa) has a disintegrin-like and cysteine-rich domain. PI and PIII were already described in *B. jararacussu* venom [32]. L-amino acid oxidases are homodimeric glycoproteins with molecular masses between 50 and 150 kDa. Its subunits are non-covalently linked, and some LAAOs were identified in *B. jararacussu* with masses between 50 and 70 kDa. In the same venom, we can find more than one type of LAAO [34]. These proteins are susceptible to dissociation under pressure; in native *B. jararacussu* venom, they were found in peaks 1N and 2N. After HHP treatment, they were eluted in peaks 2P, 2′P, and 3P. Serine proteases, which are also present in peak 1N, are enzymes with a molecular mass ranging between 26 and 67 kDa. Most SVSPs are glycoproteins, with a variable number and different glycosylation sites [35]. After pressurization, they were found in peaks 2P, 2′P, and 3P; since they are monomeric proteins, it is reasonable to infer that they change their structure, reducing volume and changing apparent molecular weight in gel filtration.

On the other hand, PLA_2_s (proteins below 20 kDa) were found exclusively in peaks 3 and 4 of the native and pressurized venom (Tables S2 and S3), suggesting that this protein was not drastically affected by HHP treatment. This is in accordance with the data presented in Figure 1, which show that HHP did not extensively compromise phospholipase activity. These data suggest that some proteins undergo dissociation under pressure, changing their eluting profile. Also, HHP treatment may induce conformational changes in some venom proteins, rendering them more susceptible to venom proteases, resulting in an altered elution pattern.

#### 2.2.2. Bis-ANS Interaction

The exposure of the hydrophobic surface of proteins can be detected using bis-ANS. This hydrophobic probe has a marginal fluorescence when free in an aqueous solution but a marked fluorescence intensity when bound to hydrophobic cavities in proteins [36]. In the absence of venom, the fluorescence quantum yield of bis-ANS is meager, as expected, but increases markedly by 8- and 76-fold in the presence of equal amounts of Bj-NV or Bj-PV, respectively (Figure S2). These results suggest that HHP, in addition to inducing the dissociation of LAOOs with the further hydrolysis of several proteins, also causes changes in the structure of the venom constituent proteins, exposing hydrophobic segments that were previously hidden.

### 2.3. Serum Production in Equines

Hereafter, both venoms (Bj-NV and Bj-PV) were used to obtain hyperimmune serum in horses. Four days after the first venom application, the horses that were injected with Bj-NV exhibited local bleeding, bloody stools, and hematuria. During the whole inoculation protocol, other local complications were observed, such as abscesses and edema at the injection site that extended to the chest and anterior abdomen. However, these symptoms were not present in horses injected with the same amount of Bj-PV (Table 1), suggesting a profound alteration in the toxic components of the venom.

### 2.4. Neutralization Capacity of Produced Hyperimmune Sera

**Figure 3:**
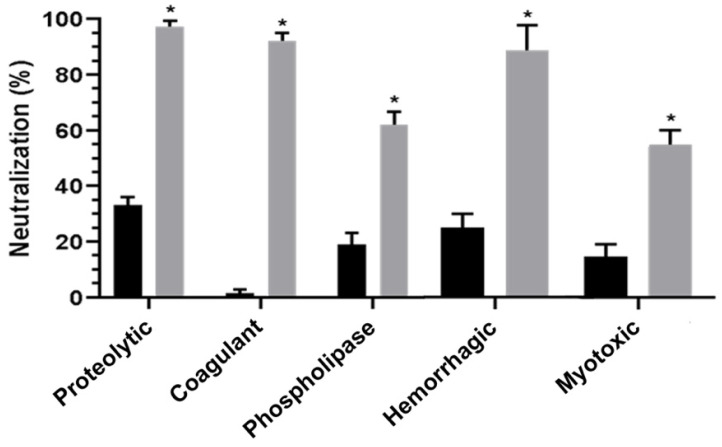
The hyperimmune sera obtained from horses injected with Bj-PV (Prejussu) presented higher neutralization activity than Bj-NV (Najussu). Aliquots of Najussu (black bar) and Prejussu (gray bar) were incubated with a fixed concentration of native venom, and the biological activities were measured. The data represent the means of three individual experiments. (* *p* < 0.05).

The serum obtained from horses injected with Bj-NV (named Najussu) and that obtained from horses injected with Bj-PV (named Prejussu) were pooled, and their immunogenicity was examined through their capacity to neutralize biological activities. We observed that Najussu failed to neutralize the coagulant activity of the *B. jararacussu* venom, while the other biological activities were neutralized by approximately 30% (Figure 3, black bars). On the other hand, Prejussu showed a high capacity to neutralize proteolytic, coagulant, and hemorrhagic activities (Figure 3, gray bars). In addition, the in vivo experiments demonstrated that Prejussu neutralized hemorrhagic and myotoxic activities by 85% and 54% (Figure 3, gray bars), while Najussu neutralized such activitites by only 20 and 15%, respectively (Figure 3, black bars).

### 2.5. Antivenomics Experiments Show That Prejussu Recognizes Proteins from Different Venoms

We applied the antivenomic technique [37] to evaluate the recognition pattern of Najussu and Prejussu toward venoms of different species and genera.

#### 2.5.1. *Bothrops jararacussu* Venom

**Figure 4:**
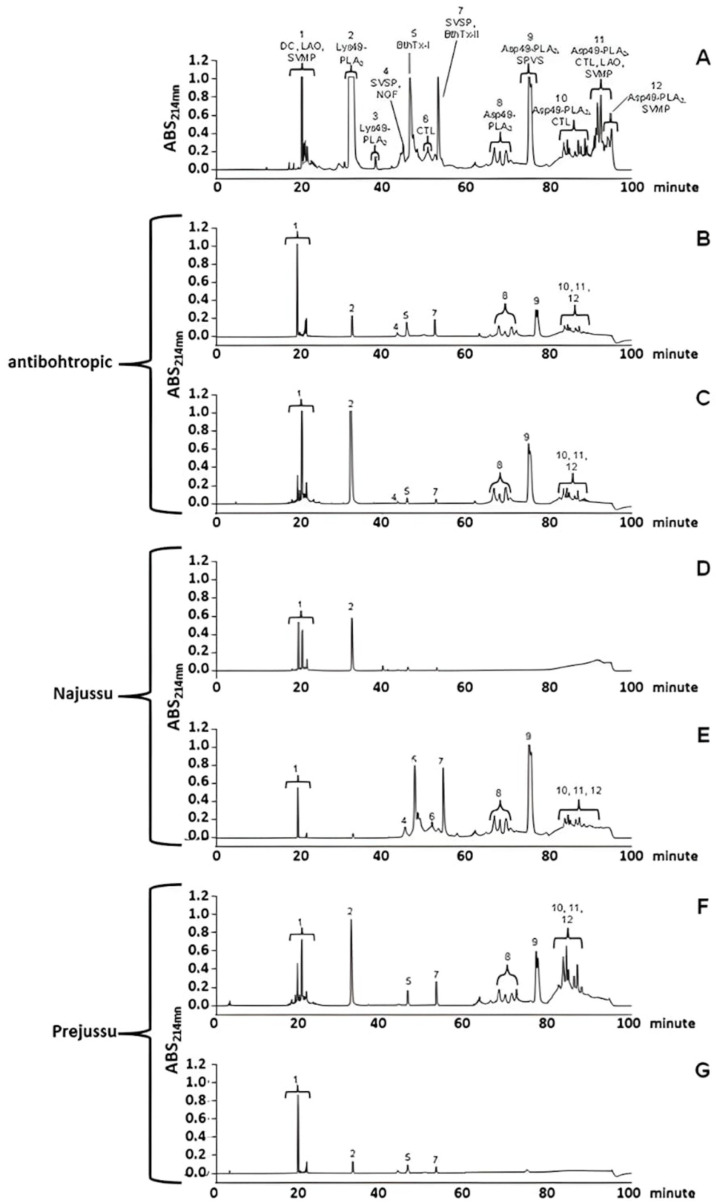
Antivenomics analysis with *B. jararacussu* venom shows that Prejussu recognizes numerous venom proteins. Affinity chromatography evaluated immunoreactivity on columns containing purified immunoglobulins from antibothropic serum, Najussu, or Prejussu. The immunocaptured and non-immunocaptured fractions of the *B. jararacussu* venom were analyzed by reverse-phase HPLC. In (**A**) crude venom profile. (**B**,**D**,**F**) proteins immunocaptured by commercial antibothropic venom, Najussu and Prejussu, respectively. In (**C**,**E**,**G**), proteins were not immunocaptured by antibothropic, Najussu, and Prejussu, respectively.

Bj-NV was fractionated by reverse-phase HPLC, followed by the identification of each protein fraction using mass spectrometry. We identified different types of proteins, such as PLA_2_, serine protease, lectin type C, metalloproteinase PI and PIII, L-amino oxidase, cysteine-rich protein, disintegrins, and NGF, which were distributed throughout the reverse-phase chromatogram (Supplemental Table S4 and Figure 4A).

First, antivenomic assays with *B. jararacussu* venom were carried out with three different sera, each one covalently bound to the resin NHS-activated Sepharose, forming three affinity columns: the first commercial antibothropic antiserum, the second Najussu, and the third Prejussu. For these experiments, the molecules of *B. jararacussu* venom that were bound and unbound to the affinity columns were identified by mass spectrometry (Figure 4). The commercial antibothropic antivenom recognized several proteins from the *B. jararacussu* venom, as seen in Figure 4B. Nevertheless, it did not recognize proteins such as PLA_2_ from peaks 2 and 9 (Figure 4C). At the same time, Najussu failed to recognize most venom toxins distributed throughout the chromatogram, recognizing only a basic PLA_2_ (peak 2) and proteins present in the first peak of the fraction (Figure 4D,E).

On the other hand, Prejussu recognized even more proteins than commercial antibothropic antiserum. This recognition was more efficient, as can be seen by the bound and unbound fractions in the chromatograms in Figure 4F,G. The proteins recognized by Prejussu were myotoxins, PLA_2_, as BthTx-I and II [27,38], and other PLA_2_ that hydrolyze cell membrane phospholipids [39]. In addition, Prejussu also recognized metalloproteinases, types PI and PIII, which are known to have high hemorrhagic activity [40]; serine proteases that affect the coagulation cascade [35]; L-amino oxidases that are associated with local and systemic effects [41]; and lectin type C, which has a potent cytotoxic effect. These data indicate increased immunogenic response to Bj-PV (Figure 4F).

In summary, Prejussu showed a great capacity to recognize a large diversity of proteins in *B. jararacussu* venom, rendering this serum a promising preparation for medical use. Its performance was even higher than that observed for commercial antibotropic venom or Najussu (serum raised against the native venom).

#### 2.5.2. Cross-Reactivity of Prejussu Antivenom

To gain more information on the Najussu and Prejussu antivenoms, we verified the existence of reactivity with other venoms from snakes of the same genus with different characteristics. In this context, we analyzed the reactivity of Najussu and Prejussu against the venom of *B. jararaca*. We used the venomic of *B. jararaca* [42] as a reference for comparison with our antivenomic results. The antivenomic experiments revealed that Najussu partially recognized proteins such as Jararhagin, Jarastatin, and Bradykinin-potentiating-peptides. However, Najussu failed to recognize other toxins in the venom of *B. jararaca* (Figure S3D,E). In contrast, Prejussu showed cross-reactivity with all toxins, including metalloproteases, which have a high hemorrhagic action (Figure S3F). For comparative purposes, we also tested the commercial antibothropic serum, which was shown to recognize toxins from the *B. jararaca* venom like Prejussu (Figure S3B,C).

To check whether the cross-reaction could extend to any other genus, we tested the recognition of proteins from the South American rattlesnake *Crotalus durissus terrificus* venom (Figure S4). To determine which proteins cross-react with Najussu and Prejussu, we used the study of the *C. d. terrificus* venom [43] as a reference for comparison with our antivenomic results. We observed that Prejussu recognizes the most toxic proteins from *C. d. terrificus* venom, such as crotamine and the crotoxin complex (Figure S4D). However, Najussu failed to recognize all proteins from *C. d. terrificus* venom (Figure S4B).

### 2.6. Prejussu Shows Higher Neutralizing Potency than Najussu Against Other Venoms

**Figure 5:**
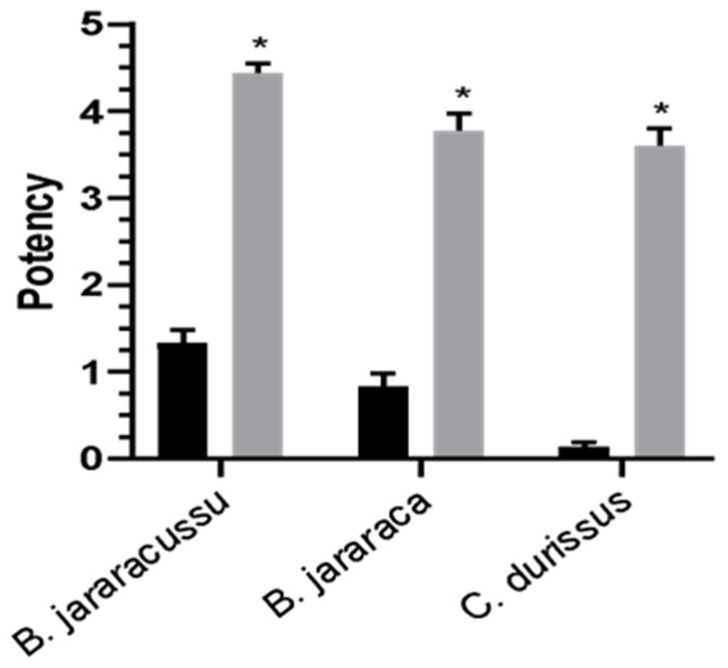
Comparison of the neutralizing potency of Najussu and Prejussu in reducing the lethality of three different venoms. The native venom of *B. jararacussu*, *B. jararaca*, and *C.d. terrifius* was incubated with doses of Najussu (black bar) and Prejussu (gray bar). This mixture was injected intraperitoneally in mice weighing 18–22 g. The animals were observed for 48 h, and the number of dead animals was used to calculate the Effective Dose, employing the statistical method of PROBIT. The serum potency is defined as the quantity of venom (in mg) neutralized by 1 mL of serum. The data represent the mean of three individual experiments. (* *p* < 0.05).

Next, we tested whether Najussu and Prejussu could neutralize the lethality of *B. jararacussu*, *B. jararaca*, and *C. d. terrificus* venoms. As observed, Prejussu had a potency approximately four-fold higher than Najussu in neutralizing all tested venoms (Figure 5)

### 2.7. ELISA Analyses Show That Prejussu Cross-Reacts with Other Venoms from the Same Genus

**Figure 6:**
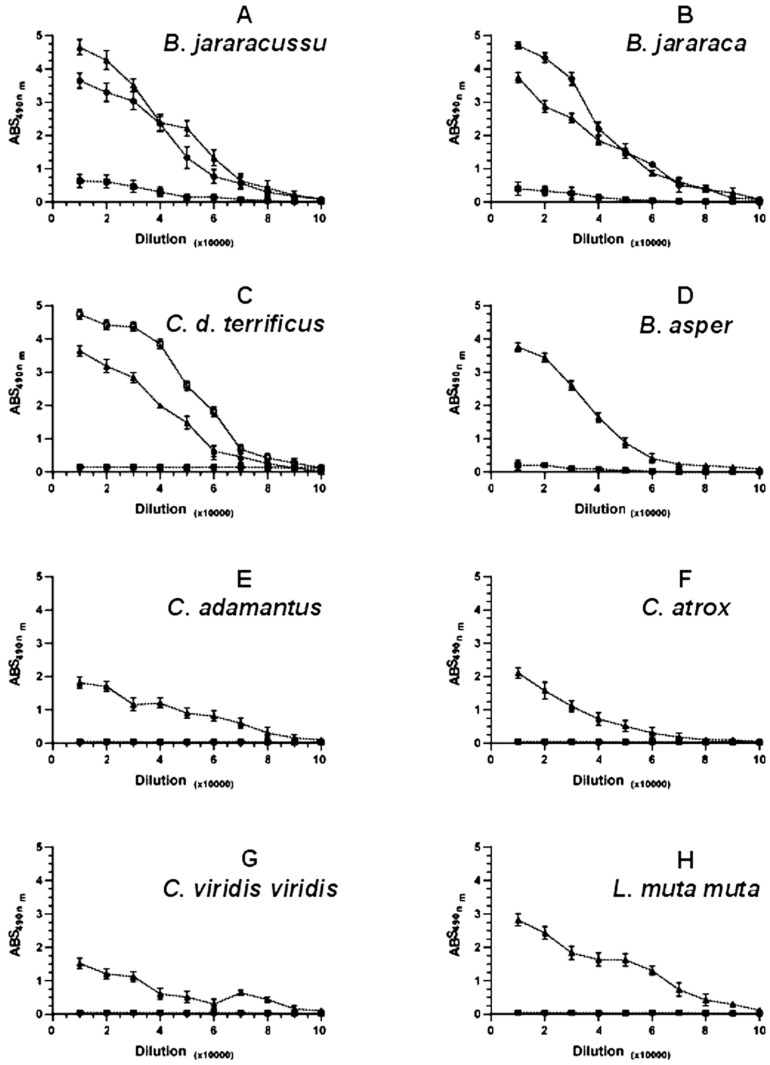
ELISA reactivity of antivenoms against different venoms. Plates were coated with 1 µg/well of each venom and incubated with commercial antibothropic sera (●), commercial anticrotralic sera (□), Najussu (■), or Prejussu (▲) antivenoms in different dilutions. The venoms used were from (**A**) *B. jararacussu*; (**B**) *B. jararaca*; (**C**) *C. durissus terrificus*; (**D**) *B. asper*; (**E**) *C. adamanteus*; (**F**) *C. atrox*; (**G**) *C. viridis viridis;* and (**H**) *L. muta muta*. Non-immune serum of horses was used as a negative control. The optical density at 490 nm determined the intensity of the reaction. The data represent the average of five individual experiments (*p* < 0.05).

Due to the unexpected cross-reactivity of Prejussu against the venoms of *B. jararaca* and especially *C. d. terrificus*, we decided to perform an ELISA analysis to identify if there is cross-reaction with other medical-relevant Viperid venoms from different regions and countries of the American continent, what would expand the use of the present methodology to other areas of the continent where the snake venom accidents are frequent. As expected, the ELISA confirmed the high reactivity of Prejussu against *B. jararacussu*, *B. jararaca*, and *C. d. terrificus*, as well as confirming the ineffectiveness of Najussu in recognizing all these venoms (Figure 6A–C). However, Prejussu also showed strong recognition for the venom of *B. asper*, a snake found in Central America and northern South America.

Other viper venoms from different Genus, such as (1) *C. adamantus*, a rattlesnake endemic to the southeastern United States of America, (2) *C. atrox*, a rattlesnake from the United States of America and Mexico, (3) *C. viridis viridis*, a rattlesnake present in Canada, the United States of America, and Mexico, and (4) the venom of *L. muta muta*, considered the largest venomous snake in Latin America, it is found preferentially in forests, including the Amazon and Atlantic Forests, were also recognized by Prejussu but with mild recognition. Again, Najussu could not recognize venoms from North, Central, and South American snakes (Figure 6D–H).

## 3. Discussion

Since the introduction of hyperimmune sera in 1894 by Physallyx, Berthrand, and Calmete {for more details, see [44], the intravenous administration of antivenoms derived from serum of animal origin has become the only therapeutic option for the treatment of envenoming by venomous animals [1,45]. Currently, several public and private laboratories around the world manufacture these antivenoms. However, several characteristics of their manufacturing process may restrict their clinical efficacy due to the high toxicity and low immunogenicity of certain toxins from venoms, which limit the injection of appropriate doses to obtain a satisfactory immune response [13,46]. Studies on the effectiveness of antivenoms produced today have emphasized a discrepancy between their toxicity and immunogenicity [13,46,47,48]. Many immunogenic components of the venom are irrelevant to their general toxicity, while other highly toxic components are poorly immunogenic or even immunosuppressive [47,48,49]. For this reason, antivenoms may be unable to effectively neutralize the venom for which they are intended [13,50].

In addition, immunization protocols using venom have changed little in over a century of production. Therefore, studies that may contribute to a better understanding of these antigens’ role in producing high-affinity antibodies that are nontoxic to experimental animals are of interest. In this context, new experimental strategies are being introduced to obtain low-toxicity and higher-immunogenicity antigens. These procedures include four alternative venom-dependent immunization approaches: (1) the use of liposomes as a delivery system [10,51,52]; (2) polymeric nanoparticles of chitosan or calcium alginate [53,54]; (3) chemical detoxification with formaldehyde [17], glutaraldehyde [11], alkylation [15,55], or iodination [56]; and (4) physical detoxification using irradiation [16], gamma radiation [57], or ultra-violet light [58]. It is noteworthy that, in recent years, new innovative immunization strategies independent of the use of venom have also been developed to improve the production of antivenoms, such as recombinant toxins and epitopes of synthetic peptides [59]. Such strategies demonstrated the potential to generate antivenoms with high titers of therapeutic antibodies and broad neutralizing activity [59,60]. Furthermore, they eliminate the dependence on venoms and animal manipulation to produce high-quality antivenoms [59,60,61,62,63]. Nevertheless, these new approaches have a long way to go before being used in patients.

Here, we used HHP to inactivate the *B. jararacussu* venom and obtain hyperimmune serum in horses. The ideal condition for treating *B. jararacussu* crude venom was established (600 MPa/24 h/37 °C). Pressures from 500 to 1000 MPa induce oligomeric protein dissociation, leading to monomeric, partially denatured states and exposing new epitopes [64]. Proteins’ tertiary/quaternary structures are mainly maintained by van der Waals interactions, salt bridges, and hydrophobic interactions, which are all susceptible to HHP [64]. In addition, after decompression, although several proteins recover their native states, several others assume a “native-like” conformation, with internal patches still exposed to the solvent. The immune system could “see” these novel antigenic sites, leading to a more robust immunogenic response. This phenomenon has already been described for isolated proteins, such as transthyretin [65], hemoglobin of *Glossoscolex paulistus* [66], gastropod hemocyanin [67], viral capsids [68,69], or even monomeric proteins [64], and was explained to be a result of a process known as “conformational drift”, in which the atomic contacts are replaced by contacts with the solvent [70,71].

In the case of *B. jararacussu* venom, HHP treatment totally inactivated the coagulant, proteolytic, and hemorrhagic activities, as well as partially inactivating phospholipase and myotoxic activities (Figure 1). The pressurized venom (Bj-PV) was two times less toxic than the non-pressurized one. We hypothesize that the lack of complete inactivation of PLA_2_ molecules may be related to their stability, since they have seven disulfide bonds and a calcium binding site that contributes to the maintenance of the tertiary structure and enzymatic site [25,72,73], thus also limiting the appearance of new epitopes. This was corroborated via gel filtration of pressurized venom results and the limited neutralization of Prejussu toward phospholipase and myotoxic activity.

The chromatographic profile of the pressurized venom (Figure 2B) shows that some proteins dissociate and undergo changes in their apparent hydrodynamic volume. In contrast, others are hydrolyzed, probably through exposing new scissile bonds. The decrease in proteolytic, coagulant, and hemorrhagic activities that was observed was possibly caused by protein hydrolysis and/or changes in protein conformation.

Several studies with proteases and HHP have been performed in the last few years [74,75,76]. Interestingly, in some cases, HHP could activate proteolytic enzymes, and protein hydrolysis occurred faster and more efficiently under pressure [74,77]. Using milk whey proteins as a substrate and three different proteases (trypsin, chymotrypsin, and pepsin), Peñas and collaborators (2006) demonstrated that chymotrypsin and trypsin have higher proteolytic activity at 100 and 200 MPa, respectively. They also showed that pepsin only hydrolyzes whey proteins when first pressurized or when pepsin is subjected to HHP in the presence of the substrate [78]. We cannot exclude the possibility that HHP treatment at low–moderate pressures could also activate venom proteases, which are then inactivated at higher pressures.

In the present study, untreated and pressurized *B. jararacussu* venom was used to obtain the hyperimmune sera from equines. Animals inoculated with pressurized venom presented no injury (Table 1). Such protection was previously observed for scorpion venom toxin treated with formaldehyde [17] or venom treated with glutaraldehyde [11] or modified by iodination [14,56], as well as for *B. jararacussu* venom chemically modified via p-bromophenacyl bromide [15]. However, these chemical detoxifications did not induce a satisfactory immune response and the antibody titers were not changed. Nonetheless, when chemically modified venoms stabilized within liposomes were injected into the horses, venom toxicity was abolished, and an increase of at least 30% in the antibody titer was observed, which persisted for several days after immunization [51]. It is worth emphasizing that the protocol we present does not include the addition of any chemical modifier or liposomes, which means that it does not increase production costs.

Comparing both sera, Prejussu was more efficient than Najussu in reducing the toxic effects of native venom (Figure 3). These results suggest that the hydrolysis of proteins and the exposure of hydrophobic segments induced by pressure (Figure S2) resulted in new epitopes that allowed for the generation of more specific antibodies to neutralize several toxic effects of untreated venom; this was corroborated by antivenomic assays. It is important to note that Najussu failed to neutralize the hemorrhagic activity of the *B. jararacussu* venom, as well as failing to recognize the metalloproteinases of the *B. jararacussu* and *B. jararaca* venoms in the antivenom experiments (Figure 3, Figure 4D and Figure S3D).

Najussu showed low reactivity with the *B. jararacussu* venom (Figure 4), confirming other authors’ reports regarding this venom’s weak immunogenicity [29,31,79,80,81]. Prejussu also showed high cross-reactivity with *B. jararaca* venom (Figure S3F) and *C. durissus terrificus* (Figure S4F), mainly against crotamine, acidic, and basic crotoxins. Crotoxin corresponds to the most significant fraction of this venom, being considered the most toxic portion [82]. This fraction consists of two subunits, a basic (PLA_2_) and an acidic one (crotapotine), which enhance the lethality of PLA_2_ [82]. The increase in the cross-reactivity of Prejussu reinforces the brilliant idea of Vital Brazil regarding *B. jararacussu* venom having similar properties for both the Bothrops and Crotalus genus [83].

Furthermore, the ELISA also showed that this high recognition range extends to viper venoms other than *B. jararaca* and *C. d. terrificus,* with a distinct geographical distribution and different envenomation symptoms than those caused by *B. jararacussu* (Figure 6). Still, in this context, previous studies have already demonstrated the successful effects of HHP in attenuating virulence and increasing the immunogenicity of viruses [18,19,20,84]. These HHP viral inactivation treatments abolished virus infectivity in mice and exhibited complete protection when the animals were challenged with native viruses [18,19,20,84]. Furthermore, viruses treated with HHP can expose previously hidden epitopes, further increasing immunogenicity [85]. Given these observations and based on the results obtained here, applying HHP in the production of antivenoms would be a promising tool for improving serotherapy against snake bites. In future studies, we intend to broaden these cross-reactivity analyses to include other relevant venoms, such as *B. atrox*, from the Amazonian region. *B. atrox’*s venom shows significant variability [86,87] and could benefit from broader recognition as an antivenom.

It is worth emphasizing that implementing this technology will require overcoming challenges such as scaling up production and the associated costs, the need for regulatory approval and compliance, its acceptance and adoption by the medical community and patients, and adequate infrastructure and resources for its implementation and maintenance. However, the potential benefits of this technology outweigh the challenges and costs.

## 4. Conclusions

In conclusion, from our study with *B. jararacussu* venom and HHP, two fundamental features have emerged: 1. the total or partial inactivation of various biological activities related to the toxicity of the venom, including a minor toxic effect on horses in which it was used for immunization; and 2. an increase in the scope of recognition of the antivenom, which began to recognize not only the venom of *B. jararacussu* but also venoms of different species of the family Viperidae and subfamily Crotalinae. These features are relevant because animals used for immunization would suffer less and could live longer, leading to the augmentation of production. Also, the hyperimmune sera could be used for different accidents with Viperidae snakes.

## 5. Materials and Methods

### 5.1. Animals

#### 5.1.1. Mice

Female Swiss mice weighing 18 to 22 g, supplied by the Animal Health Control Laboratory of the Vital Brazil Institute (IVB) or the Butantan Institute, were used in the different experiments. These animals were kept under controlled environmental conditions (light and temperature) and received water and food ad libitum. The Ethics Committee in Research of Animal Use from Butantan Institute 697/10 approved the animal use.

#### 5.1.2. Horses

Instituto Vital Brazil (IVB) has an experimental farm of 17 bushels in Cachoeiras de Macacu in Rio de Janeiro. The horses were selected on the farm, which follows all health and animal nutrition recommendations stipulated by the WHO, 2011. The inclusion criterion considered naive horses weighing between 330 kg and 550 kg, aged between 5 and 8 years.

### 5.2. Venoms and Commercial Antivenoms

The venoms of *Bothrops jararacussu* (batch 2010BJU03401) and *Bothrops jararaca* (batch 2010BJA03514) used in this study were collected from at least thirty distinct specimens. The IVB provided both venoms. The crotamine-positive *Crotalus durissus terrificus* venom (batch 2014CDU00301) was extracted from 26 adult specimens housed at the Regional Ophiology Center of Porto Alegre (NOPA). Venoms of *Lachesis muta* and from central and North American snakes (*B. asper*, *C. atrox*, *C. adamanteus*, and *C. v. viridis)* were provided by Dr. Paulo de Assis Melo of the Federal University of Rio de Janeiro, Institute of Biomedical Sciences, Department of Basic and Clinical Pharmacology. The specific commercial antivenoms antibothropic (batch SAB165A08C) and crotamine-positive anticrotalic (batch SAC155204F) were all produced in the IVB, based on the guidelines of the Brazilian Pharmacopoeia and the instructions of ANVISA (Ministry of Health 1996). These antivenoms are of equine origin and consist of purified F (ab’) 2 fragments.

### 5.3. Treatment by HHP

*B. jararacussu* lyophilized venom was weighed, resuspended in distilled water (final concentration 10 mg/mL), and divided into two volumes that were equally distributed in distinct vacuum-sealed poly-nylon packages. Half the packages were placed in a stainless-steel vessel capable of withstanding high-pressure levels, managed by a laboratory pilot scale HHP generator (pressurized venom—Bj-PV). The other half was maintained at 1 bar (native venom—Bj-NV). All tested conditions are displayed in Supplemental Table S1. The pressure-transmitting fluid used in the HHP generator was distilled water with synthetic carboxylate-based concentrated fluid add in a proportion of 2:1 (*v*/*v*), following the manufacturer’s instructions. The control and pressurized samples were aliquot (1 mL per tube) and stored at −20 °C.

### 5.4. Bis-ANS Fluorescence

*B. jararacussu* venom (1 mg/mL) was maintained at atmospheric pressure (Bj-NV) or submitted to 600 MPa for 24 h at 37 °C (Bj-PV). After decompression, Bj-NV and Bj-PV were stored at −20 °C. Before obtaining the spectra, the samples were diluted in saline. Small volumes (7.2 μL) of bis-ANS (280 μM) were added to solutions of 2 mL of Bj-NV or Bj-PV (20 μg/mL). The final concentration of bis-ANS was 16 μM. For Bj-NV or Bj-PV, the maximum dilution at the end of the experiment was approximately 2.9%. At room temperature, the bis-ANS fluorescence spectra were recorded on a K10 PCI spectrofluorimeter (ISS Inc., Champaign, IL, USA). Bis-ANS emission was measured by exciting the samples at 360 nm and collecting the emission from 4000 through 600 nm after equilibration for 5 min.

### 5.5. Gel Filtration Chromatography

Initially, 10 mg of Bj-NV and Bj-PV were diluted in PBS (50 mM sodium phosphate, 0.15 M sodium chloride, pH 7.4). Then, Bj-NV and Bj-PV were centrifuged at 10,000× *g* for 10 min. The supernatant was applied to a Superose 12 column (10/300 GL) previously equilibrated with a buffer containing the same dilution of the venoms at a flow of 1 mL/min at room temperature, and the fractions were collected in 1 mL aliquots. The elution of the samples was monitored through absorbance at 214 nm.

### 5.6. SDS-PAGE

Bj-NV and Bj-PV were fractionated in 12.5% polyacrylamide gel in the presence of SDS, as described by Laemmili, 1970 [88]. Bj-NV and Bj-PV were mixed with sample buffer (Tris-HCl 12 mM, pH 6.8, 20% glycerol, 0.5% SDS, EDTA 0.1 M, and 0.05% Bromophenol Blue) and boiled for 5 min and before being placed (20 μg per well) in a 4% polyacrylamide stacking gel. The running buffer contained Tris 25 mM, glycine 0.19 M, and 0.1% SDS, pH 8.3. Electrophoresis was performed at room temperature, using a constant-amperage electric field (20 mA) and voltage of around 200 V. Gels were stained using Coomassie Blue (0.5% Coomassie R-250 Blue, 45% methanol, and 10% acetic acid). Gels were destained with a solution of 37% methanol and 10% acetic acid. The densitometry of the gels was analyzed by ImageJ.

### 5.7. Biological Activities

#### 5.7.1. Proteolytic Activity

It was determined using an azocasein solution as substrate. Bj-NV or Bj-PV (80 µg/mL, 275 µL) were incubated with a 0.5% azocasein (225 µL) solution containing 10 mM Tris-HCl and 100 mM NaCl for 30 min at 37 °C. After this interval, the reaction was stopped by adding 500 µL of 15% trichloroacetic acid (TCA), the tubes were centrifuged at 16,443× *g* for 10 min, and the supernatant (1 mL) was mixed with NaOH (0.5 M, 500 µL). The same procedure was used as a negative control without the venom solution. The absorbance of each fraction was read at 420 nm.

#### 5.7.2. Coagulant Activity

The ability of Bj-NV or Bj-PV to potentiate blood coagulation in vitro was evaluated by plasma recalcification assay according to the following protocol: native and pressurized *B. jararacussu* venom (50 µL, 4.4 ng/mL) diluted in Tris-buffered saline (pH 7.4) were incubated with 50 μL of platelet-poor citrated human plasma for 1 min at 37 °C. Then, 25 mM CaCl_2_ (100 μL) was added, and the time required for coagulation was monitored on an Amelung KC-4 Coagulometer (Amelung Ltd., Labcon, Germany). The same procedure was used as a negative control, excluding the addition of venom. The use of human blood was approved by the Ethics Committee in Research of the University Hospital Clementino Fraga Filho-UFRJ (Opinion Number: 1,399,569).

#### 5.7.3. Phospholipase Activity

Phospholipase activity was determined using phosphatidylcholine as a substrate. A solution containing 0.15 M NaCl, 10 mM CaCl_2,_ and 0.5% (*v*/*v*) Triton X-100 was prepared, and 0.3 g phosphatidylcholine, at a final concentration of 2.5% (*w*/*v*), was added to 12 mL of this solution. The solution was sonicated for 1 h to dissolve the phosphatidylcholine completely. To finalize the reagent solution, 240 µL of 0.15% (*w*/*v*) phenol red diluted in 1 mM NaOH was added. Then, in each tube containing 2.5 mL of this reagent solution, 100 µL (0.2 µg/µL) of Bj-NV or Bj-PV was added. For negative control, venom was substituted with 100 µL of distilled water. The tubes were incubated at 37 °C for 1 h and then centrifuged for 5 min at 5000 g in a single bench centrifuge at room temperature. The absorbance of the supernatant was read at 540 nm. The same procedure was used as a positive control, replacing the venom with a lecithin solution. 1U corresponds to the amount of venom (µg venom/assay) that decreases 0.05 the absorbance units.

#### 5.7.4. Hemorrhagic Activity

Hemorrhagic activity was determined according to [89], with some modifications. The activity was estimated visually on the dorsal skin of mice. For this, samples containing 200 μg of Bj-NV or Bj-PV were injected intradermally into the back of mice. After 3 h, the animals were euthanized in a CO_2_ chamber, and the dorsal skin was removed to evaluate the hemorrhagic area. *B. jararaca* crude venom was used as a positive control because it is highly hemorrhagic. The hemorrhagic activity was estimated considering the product between the lesion area’s most extensive and minor diameter (cm^2^).

#### 5.7.5. Myotoxic Activity

Groups of mice (*n* = 6) received, in a single point at the right gastrocnemius muscle, 50 μg (50 μL/animal) of Bj-NV or Bj-PV diluted in sterile saline. One group of mice received 50 μL of sterile saline as a control. After 4 h, six mice from each group were bled to obtain plasmas and detect creatine kinase levels using the UV kinetic CK (BioClin, Belo Horizonte, Brazil).

#### 5.7.6. Lethal Dose 50%

The protocol used to determine the 50% lethal dose (LD_50_) was based on that indicated by Brazil (Ministry of Health 1996). Thus, the venoms were diluted in saline (0.85%) at a concentration of 1 mg/mL (stock). Immediately afterward, increasing volumes of the stock solution were distributed, maintaining a dilution factor of a maximum of 1.5 in separate tubes, and saline was added to obtain the desired doses for the native (50, 75, 112, and 160 µg) and pressurized (112, 160, 240 and 360 µg) venoms. For each dose, a group of 8 mice weighing 17–22 g was used, and a constant volume of 0.5 mL was inoculated via the intraperitoneal route. Mice were observed for 48 h, and the number of dead was used to calculate the LD_50_ using the PROBIT statistical method.

### 5.8. Mass Spectrometry

The mass spectra of the analyzed samples were obtained using the quadrupole/orthogonal Q-Tof spectrometer (Waters, Milford, MA, USA) with an interface to the Nano Acquity capillary chromatography system. Electrospray ionization (ESI) was performed using 3500 V, with a source temperature of 80 °C and a cone voltage of 30 V. The data acquisition and control of the instrument were carried out in the MassLynx program (Version 4.1, Waters). Chromatographic runs were performed with an amplitude of 400–2000 mass/charge ratio (*m*/*z*), using 1 s intervals applied throughout the chromatographic process. The data-dependent acquisition of MS/MS fragmentation was performed on precursors with loads 2, 3, and 4 throughout 50–2000 (*m*/*z*) with an interval of 2 *m*/*z*. A maximum of 3 ions were selected from each spectrum (MS) for further fragmentation by MS/MS. The collision-induced dissociation (CID) was obtained using argon gas at a pressure of 40 psi and a collision energy ranging from 18 to 90 V, depending on the precursor load and mass. All data were processed using the ProteinLynx Global server (version 2.5, Waters), where the value of the mass/charge of each precursor (MS) and fragment (MS/MS) was determined from the mass spectra coming from these chromatograms, using the Q-Tof LockSpray™ system (Waters, Milford, MA, USA) for calibration.

### 5.9. Immune Serum Produced in Horses

Six horses, selected for the production of hyperimmune serum, were divided into two groups (*n* = 3) that were named according to the immunization received: native venom of *B. jararacussu* (Bj-NV) and pressurized venom of *B. jararacussu* (Bj-PV) (CEUA under protocol number 007/2015). Both groups underwent a primary immunization that consisted of eight inoculations of the corresponding venom antigen for each group (Bj-NV or Bj-PV) with an interval of one week between them; for the first immunization, 5 mg of antigen diluted in 5 mL of sterile saline and 5 mL of complete Freund’s adjuvant were applied. The second and third immunizations were like the first, with the complete Freund adjuvant being replaced with the same volume of montanide adjuvant ISA50V2. In the other injections (4 to 8), 5 mg of antigen, diluted in 10 mL of sterile saline, was applied. After 30 days of the last primary immunization, the horses were re-immunized two more times, with a 30 day interval between each immunization. Each reimmunization consisted of 4 applications of antigen in doses of 7.5 mg (first applications) and 2.5 mg (other applications), diluted in 10 mL of sterile saline with an interval of seven days between the first and the second reimmunization and 48 h between the second/third and third/fourth. Seven days after immunization, a sample of each animal’s plasma was taken to analyze the immunization via ELISA. After 14 days, blood was taken, plasma were pooled by group, and the neutralization and potency were measured. Each group of animals showed similar responses. Fourteen days after the end of the immunizations, blood collection was carried out in a double bag, in a closed circuit, with direct passage to the interior and ensuring the area was free of contamination; 6 L of blood was collected from each animal.

The bags were sealed and stored for 48 h at 4–8 °C to promote red cell settling. Then, the bags were placed in the plasma extractor, and the hyperimmune plasma was transferred to the final bag, which was stored at 4–8 °C until the moment of use. The plasma elements were resuspended in 2 L of 0.9% sodium chloride solution and reinfused in the respective horse from which it was collected. Subsequently, the plasma bags had their immunoglobulins G purified for the performance of antivenom tests, potency tests, and ELISA. The serums named Najussu and Prejussu are, respectively, the serums obtained from immunizations with the Bj-NV and Bj-PV.

### 5.10. Neutralization of Biological Activities

Using the produced sera, biological activities were neutralized. To this end, each serum or purified immunoglobulin was incubated for 30 min with *B. jararacussu* venom at the concentrations described in the text and assayed for the corresponding biological activities (proteolytic, coagulant, phospholipase, hemorrhagic, and myotoxic).

### 5.11. Purification of Immunoglobulins G

Serums were delipidated by adding 20 µL/mL of a 10% dextran sulfate solution and 0.1 mL/mL of a 1 M calcium chloride solution. The mixture was stirred for 15 min and centrifuged at 5000× *g* for 10 min. The supernatant corresponding to the lipid-free serum was dialyzed against PBS and applied to a protein G-Sepharose column affinity chromatography that was previously balanced with the same dialysis buffer. Unabsorbed proteins were removed by washing the column with PBS. Column-bound immunoglobulins were eluted with 0.2 M glycine buffer, pH 2.8. The chromatographic profile was monitored on a 280 nm UV (ultraviolet) monitor (EM-1 Econo UV Monitor Biorad, Hercules, CA, USA) with a sensitivity of 1.0. The eluates were concentrated and dialyzed against the PBS. The antibody solution was stored at −20 °C.

### 5.12. Antivenomics

The antivenomics experiments were based on the methodology described by Pla, Gutiérrez, and Calvete, 2012 [37]. A total of 1 mL NHS-activated Sepharose 4 Fast Flow (GE Healthcare) was packaged in 4 columns and washed with 10–15 matrix volumes with 1 mM HCl, followed by two volumes of coupling buffer (0.2 M NaHCO_3_, 0.5 M NaCl, pH 8.3) to adjust the column pH to between 7.0 and 8.0. Then, 50 mg of the antibodies isolated from the commercial antibothropic and anticrotalic serums or the prepared sera (Najussu and Prejussu) were incubated with the matrix for 4 h at room temperature. Coupled IgG molecules were quantified by SDS-PAGE band densitometry (Image J 1.46 software). Coupling yields were 21 mg for antibothropic, 19 mg for anticrotalic, 23 mg for Najussu, and 22 mg for Prejussu. Unreacted functional groups with the matrix were blocked through incubation with 1 mL of 0.1 M Tris-HCl, pH 8.0, for 20 h on an orbital shaker. The affinity columns were washed alternately with three volumes of 0.1 M acetate/0.5 M NaCl, pH 4.0–5.0, and 0.1 M Tris-HCl, pH 8.5 buffers. This treatment was repeated six times. Before incubation with venoms, the matrix was equilibrated with five volumes of PBS. A total of 500 µg of *B. jararacussu*, *B. jararaca*, and *C. terrificus* venoms was incubated with antivenom (antibody) for the immunoaffinity assay. The antivenom ratios were as follows: venom was 42:1 for antibothropic, 38:1 for anticrotalic, 46:1 for Najussu, and 44:1 for Pressuju. These compositions were dissolved in ½ volume PBS and incubated for 4 h with the matrix at 25 °C using an orbital shaker. As controls, 200 µL of NHS-activated Sepharose 4 Fast Flow without or with 8.5 mg of immobilized (preimmune) IgGs was incubated with the venoms, and the columns were developed in parallel with the immunoaffinity experiment. After the collection of unbound fractions, the columns were washed five times with PBS, and the immunocaptured venom proteins were eluted with five volumes of elution buffer (0.1 M glycine, pH 2.0) and neutralized with 1 M Tris-HCl, pH 9.0. The non-retained and immunocaptured venom fractions were fractionated by reverse-phase HPLC using a C18 column. The flow was adjusted to 1 mL/min, and the column was developed with a linear gradient, as described by [37] Protein detection was performed at 214 nm.

### 5.13. Serum Potency

The protocol used for serum potency determination was an effective dose of 50% (ED_50_), as the World Health Organization indicated in 2013 [90] (CEUA #007/2015). A fixed amount, five times the LD_50_ value of the native *B. jararacussu*, *B. jararaca*, and *C. durissus* venoms, was incubated with varying Najussu and Prejussu sera doses for 30 min at 37° C. Each mixture (0.5 mL) was injected intraperitoneally into groups of 8 Swiss mice (18–22 g). Mice were observed for 48 h, and the death counts were used to calculate ED_50_ using the PROBIT statistical method. Serum potency is defined as the amount of venom, in milligrams, neutralized by 1 mL of serum.

### 5.14. ELISA Assays

ELISA was performed as described by [91], with some modifications. Ninety-six-well microtiter plates (NUNC^®^/Maxisorp Waltham, MA, USA) were sensitized for 18 h at 4 °C in a humid chamber with 100 μL per well of a solution containing 10 μg/mL of the different studied venoms, each one diluted in sodium carbonate buffer, pH 9.6. After this, the plates were washed with PBS containing 0.1% Tween 20 (Sigma^®^ St. Louis, MO, USA) and then blocked for 2 h at 37 °C with a 3% BSA PBS solution (200 µL per well). After a new wash cycle, the plates were incubated for 1 h at 37 °C in a humid chamber with 100 μL per well with Najussu and Prejussu, commercial antibothropic or anticrotalic sera produced by the Vital Brazil Institute. The negative control was 1:1000 non-immune horse serums in PBS. The plates were then washed with PBS/Tween and then incubated for 1 h at 37 °C with the imunoenzymatic conjugate peroxidase-labeled horse anti-IgG enzyme (Sigma^®^), 1:10,000, diluted in PBS (100 μL/well). After a new wash cycle, the reaction was revealed through the addition of 100 μL of the substrate (1 mg OPD-o-Phenylenediamine/mL in 0.2 M citrate buffer, pH 5.0, plus 0.06% H_2_O_2_) per well. The reaction was stopped by adding 30% H_2_SO_4_ (50 μL/well). The intensity of the reaction was determined by reading the optical density at 490 nm.

### 5.15. Statistics

All statistical analyses were performed using GraphPad Prism 8.0 for Windows (GraphPad Software Inc., San Diego, CA, USA). Student’s unpaired T-tests were conducted to determine the differences between treatments. All values in the figures are presented as mean ± SEM. Statistical significance was set at *p* ≤ 0.05.

## 6. Patents

The findings of this study were crucial in the development a significant innovation in the field of antivenoms, resulting in the filing of a patent. The methodology for venom inactivation through high hydrostatic pressure, combined with advancements in the production of hyperimmune sera, was formalized under the title: “Process of total or partial detoxification of venoms from venomous animals through high hydrostatic pressure for use in the production of hyperimmune serum.” This breakthrough led to the registration of patent nº BR 10 2020 017271 9 A2, filed with the National Institute of Industrial Property (INPI). This patent not only underscores our research’s originality and practical applicability but also highlights its contribution to technological advancement and the improvement of antivenom therapies. The intellectual protection of this innovation ensures the potential for future commercial applications and enhances the dissemination of the benefits derived from this work.

## Figures and Tables

**Table 1:** Toxic effects observed after immunizations. Three horses were injected with Bj-NV and three with Bj-PV. The complications of the procedure were noted four days after the first immunization and did not change after the complete immunization process.

The Severity of the Immunizations in the Equines						
	With Native Venom (Bj-NV)			With Pressurized Venom (Bj-PV)		
	Equine 1	Equine 2	Equine 3	Equine 1	Equine 2	Equine 3
Abscesses	++	+++	++++	-	-	-
Edemas	++	++	++++	-	-	-
Local bleeding	+++	+++	+++	-	-	-
Feces/urine With blood	++	++	++	-	-	-

++ medium; +++ serious; ++++ very serious - without collateral damage.

## Data Availability

The original contributions presented in this study are included in the article/Supplementary Materials. Further inquiries can be directed to the corresponding author.

## Supplementary Materials

The following supporting information can be downloaded at: https://www.mdpi.com/article/10.3390/toxins17020088/s1, Table S1: Treament conditions for *B. jararacussu* venom; Figure S1: HHP-induced changes in the gel filtration profile; Figure S2: Evaluation of bis-ANS binding capacity in native or pressurized venom of *B. jararacussu*; Table S2: Proteins identified in the Gel filtration chromatography of Bj-NV; Table S3: Proteins identified in the Gel filtration chromatography of Bj-PV; Table S4: Reverse phase—fractions of *B. jararacussu* venom; Figure S3: Antivenomic of *B. jararaca* venom; Figure S4: Antivenomic of *C. durissus* venom.
