# Supplementary material for: Bothrops jararacussu Venom Inactivated by High Hydrostatic Pressure Enhances the Immunogenicity Response in Horses and Triggers Unexpected Cross-Reactivity with Other Snake Venoms
Source: Toxins (Basel). 2025 Feb 13;17(2):88. doi: 10.3390/toxins17020088 (PMC11861346; doi:10.3390/toxins17020088)
Supplement: Supplementary file 1 [file toxins-17-00088-s001.zip › toxins-3428805-supplementary.pdf]

Bothrops jararacussu venom inactivated by high hydrostatic pressure enhances the immunogenicity response in horses and triggers unexpected cross-reactivity with other snake venoms

Ricardo Teixeira Araújo[1, 2]; Marisa Carvalho Suarez[3]; Carlos Correa-Netto[1, 2]; Luiz Eduardo Ribeiro da Cunha[2]; Debora Foguel[1] and Russolina Benedeta Zingali[1].

[1] Universidade Federal do Rio de Janeiro, Instituto de Bioquímica Médica Leopoldo de Meis (IBqM) and Instituto Nacional de Ciência e Tecnologia de Biologia Estrutural e Bioi-magem (Inbeb). Rio de Janeiro, Brazil; rcdo.araujo@gmail.com, netto@bioqmed.ufrj.br, lzingali@bioqmed.ufrj, foguel@bioqmed.ufrj.br
[2] Instituto Vital Brazil, Niterói, Rio de Janeiro, Brazil; lcunha@nthink.com.br
[3] Universidade Federal do Rio de Janeiro, Campus Duque de Caxias Professor Geraldo Cidade, Duque de Caxias, Rio de Janeiro, Brasil; mcarvalhosuarez@yahoo.com.br

Table S1: Conditions of treatment used to treat *B. jararacussu* venom. Each condition was tested by varying hydrostatic pressure, pressurization time and temperature.

Pressure	*Time*	*Temperature*
50 MPa	8 hours	-10°C
50 MPa	8 hours	4°C
50 MPa	8 hours	37°C
100 MPa	8 hours	-10°C
100 MPa	8 hours	4°C
100 MPa	8 hours	37°C
290 MPa	8 hours	-10°C
290 MPa	8 hours	4°C
290 MPa	8 hours	37°C
290 MPa	24 hours	-10°C
290 MPa	24 hours	4°C
290 MPa	24 hours	37°C
600 MPa	4 hours	37°C
600 MPa	8 hours	37°C
600 MPa	24 hours	37°C

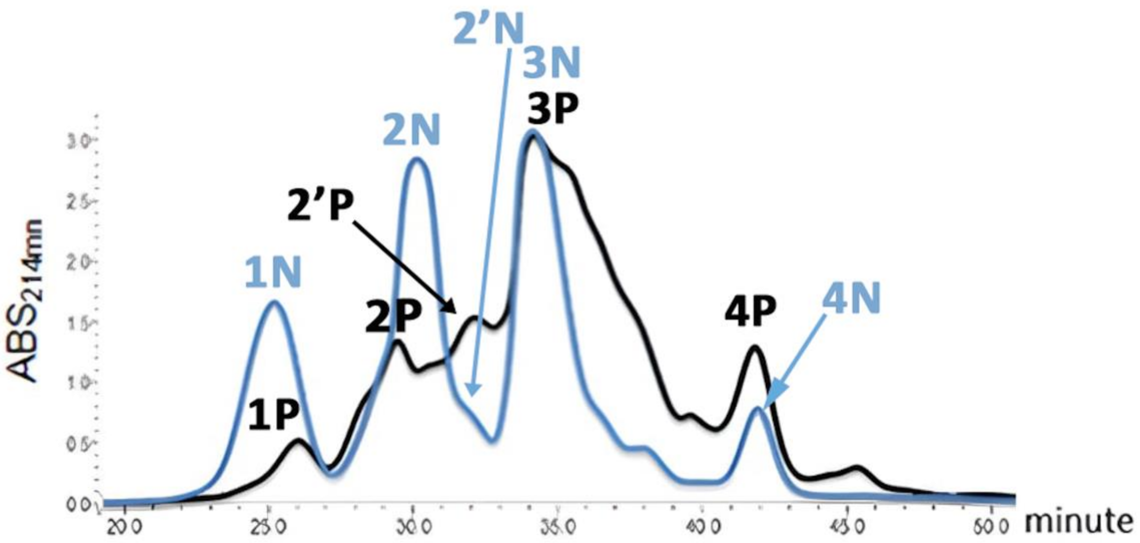

Figure S1: HHP induces changes in the gel filtration profile. 6,7 mg of *B. jararacussu* native or pressurized venom were fractionated in a Superose 12 column. Overlay of the chromatographic profiles presented *B. jararacussu* native venom (blue line) and pressurized venom (black line).

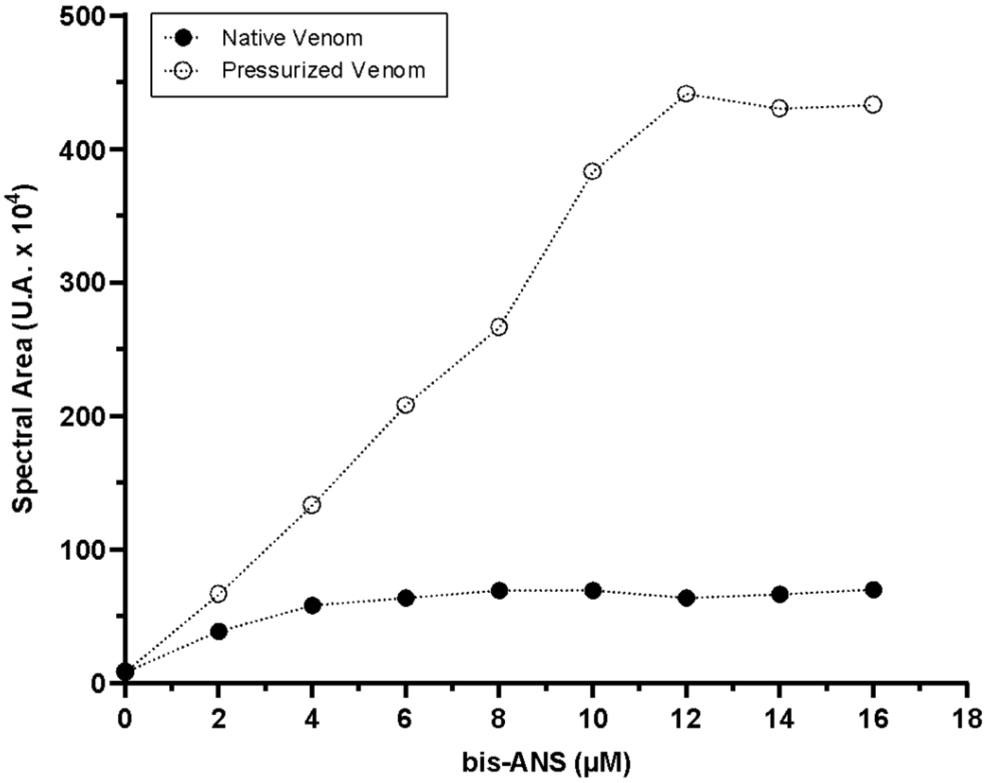

Figure S2: Evaluation of bis-ANS binding capacity in native or pressurized venom of *B. jararacussu*. bis-ANS titration with a fixed venom concentration (20 µg/ mL).

Table S2: Proteins identified in the Gel filtration chromatography of Bj-NV. List of proteins identified by mass spectrometry.

	Proteins	ID	Database	Peptides
1N	MPVS PIII	gi|32306927	NCBI	TVAPDGCFDSNQK<br>SECDIAESCTGQSPECPTDDFHR
	SPVS	gi|32396014	NCBI	VSDYTEWIR
	LAO	gi|82127389	NCBI	SAGQLYEESLQK
	LTC	gi|32396016	NCBI	DFSWEWTDR<br>EFCVELVSNTGYR
2N	MPVS PIII	gi|32306927	NCBI	TVAPDGCFDSNQK<br>SECDIAESCTGQSPECPTDDFHR
	SPVS	gi|32396014	NCBI	VSDYTEWIR
	LAO	gi|82127391	NCBI	SAGQLYEESLQK<br>VGEVNKDPGVLDYPVKPSEVGK
	LTC	gi|32396016	NCBI	NAFLCQCK<br>NQPDHYQNK<br>DFSWEWTDR
2'N	PLA_2	gi 1171973	NCBI	TIVCGENNPCLK<br>SYGAYGCNCGVLGR
3N	PLA_2	PA2H_BOTNE	SWISS PROT	SYGAYGCNCGVLGR
4N	PLA_2	PA2H_BOTNE	SWISS PROT	SYGAYGCNCGVLGR

Table S3: Proteins identified in the Gel filtration chromatography of Bj-PV. List of proteins identified by mass spectrometry.

	Proteins	ID	Database	Peptides
1P	ND	ND	ND	ND
2P	MPVS	gi|32306927	NCBI	TVAPDGCFDSNQK SECDIAESCTGQSPECPTDDFHR
	SPVS	gi|32396014	NCBI	VSDYTEWIR
	LAO	gi|82127389	NCBI	SAGQLYEESLQK
	LTC	gi|32396016	NCBI	NAFLCQCK NQPDHYQNK
2' P	MPVS PIII	gi|32306927	NCBI	TVAPDGCFDSNQK SECDIAESCTGQSPECPTDDFHR
	SPVS	gi|32396014	NCBI	VSDYTEWIR
	LAO	gi|82127389	NCBI	SAGQLYEESLQK
	PLA_2	PA2H_BOTNE	NCBI	SYGAYGCNCGVLGR
3P	MPVS PIII	Q7T1T5	NCBI	YLIDNRPPCILNIPLR
	SPVS	gi|32396014	NCBI	VSDYTEWIR
	LAO	gi|82127389	NCBI	SAGQLYEESLQK
	LTC	gi|32396016	NCBI	DFSWEWTDR EFCVELVSNTGYR
	PLA_2	PA2H_BOTNE	SWISS PROT	SYGAYGCNCGVLGR
4P	MPVS PIII	gi|32306927	NCBI	SECDIAESCTGQSPECPTDDFHR TVAPDGCFDSNQK
	PLA_2	PA2H_BOTNE	SWISS PROT	SYGAYGCNCGVLGR
	LTC	gi|32396016	NCBI	DFSWEWTDR EFCVELVSNTGYR

Table S4: Reverse phase - fractions of *B. jararacussu* venom. List of proteins identified by mass spectrometry.

	Protein	ID	Database	Peptides
Peak 1	DC	VM2I_CROBA	SWISS PROT	LRPGAQCAEGLCCDQCR<br>ARGDNPDDR<br>CTGQSADCPR
	LAO	OXLA_VIPAA	SWISS PROT	VTVLEASER<br>IQFEPPLPPK
	SVMP PI	VM2E1_PROEL	SWISS PROT	ERDLLPR<br>YNSNLNTIR
Peak 2	Lys49-PLA_2	PA2H1_BOTPI	SWISS PROT	SYGAYGCNCGVLGR<br>DATDRCCYVHK
Peak 3	Lys49-PLA_2	PA2H1_BOTPI	SWISS PROT	YHLKPFCK
Peak 4	NGF	NGFV_BOTJR	SWISS PROT	ALTMEGNQASWR
	SVSP	VSP1_BOTJA	SWISS PROT	INILDHAVCR
Peak 5	BthTx-I	PA2B1_BOTJR	SWISS PROT	MILQETGKNPAK<br>SYGAYGCNCGVLGR
Peak 6	CTL	LECG_AGKPI	SWISS PROT	NAFLCQCKF
Peak 7	BthTx-II	PA2A_BOTPC	SWISS PROT	YWFYGAK
	SVSP	VSP1_BOTJA	SWISS PROT	AAYPELPAEYR
Peak 8	Asp49-PLA_2	PA2A_BOTPC	SWISS PROT	YWFYGAK<br>CCFVHDCCYGK
Peak 9	SVSP	VSPL_BOTAS	SWISS PROT	INILDHAVCR
	Asp49-PLA_2	PA2A_BOTPC	SWISS PROT	YWFYGAK
Peak 10	Asp49-PLA_2	PA2A_BOTPC	SWISS PROT	YWFYGAK<br>CCFVHDCCYGK
	CTL	LECG_AGKPI	SWISS PROT	DFSWEWTDR
Peak 11	LAO	OXLA_BOTJR	SWISS PROT	SAGQLYEESLQK<br>EEIQAICRPSMIQR
	SVMP - PIII	VM1_BOTPI	SWISS PROT	YLIDNRPPCILNIPLR<br>SECDIAESCTGQSPECPTDDFHR
	Asp49-PLA_2	PA2A_BOTPC	SWISS PROT	YWFYGAK<br>CCFVHDCCYGK
	CTL	LECG_AGKPI	SWISS PROT	EFCVELVSNTGYR
Peak 12	SVMP - PIII	VM1_BOTPI	SWISS PROT	YLIDNRPPCILNIPLR
	Asp49-PLA_2	PA2A_BOTPC	SWISS PROT	YWFYGAK

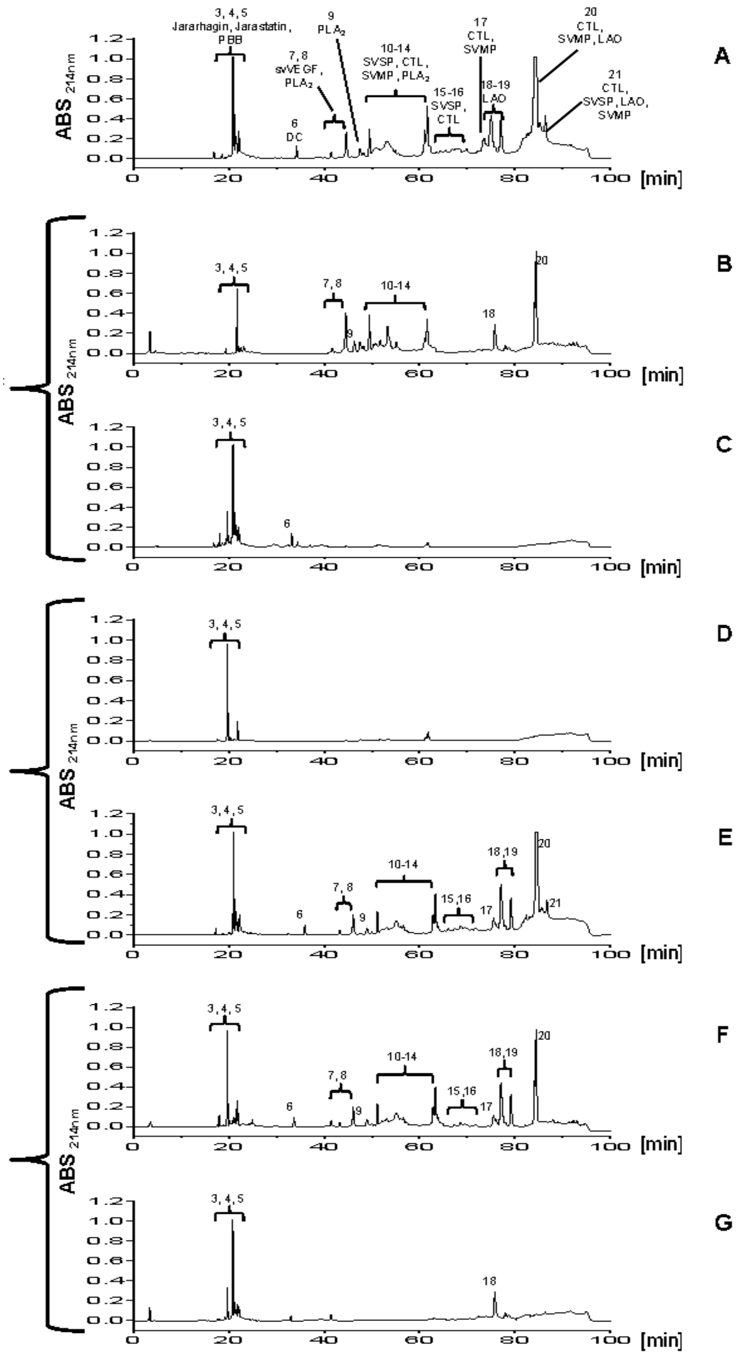

Figure S3: Antivenomic of *B. jararaca* venom. Immunoreactivity was assessed by affinity chromatography on columns containing the purified immunoglobulins from antibothropic, Najussu or Prejussu. The immunocaptured and non-immunocaptured fractions of the *B. jararaca* venom were analyzed by reverse-phase HPLC. In (A) crude venom profile, (B) proteins immunocaptured by antibothropic serum, (C) proteins not immunocaptured by antibothropic, (D) proteins immunocaptured by Najussu; (E) proteins not immunocaptured by Najussu; (F) proteins immunocaptured by Prejussu and in (G) non-immune proteins captured by Prejussu.

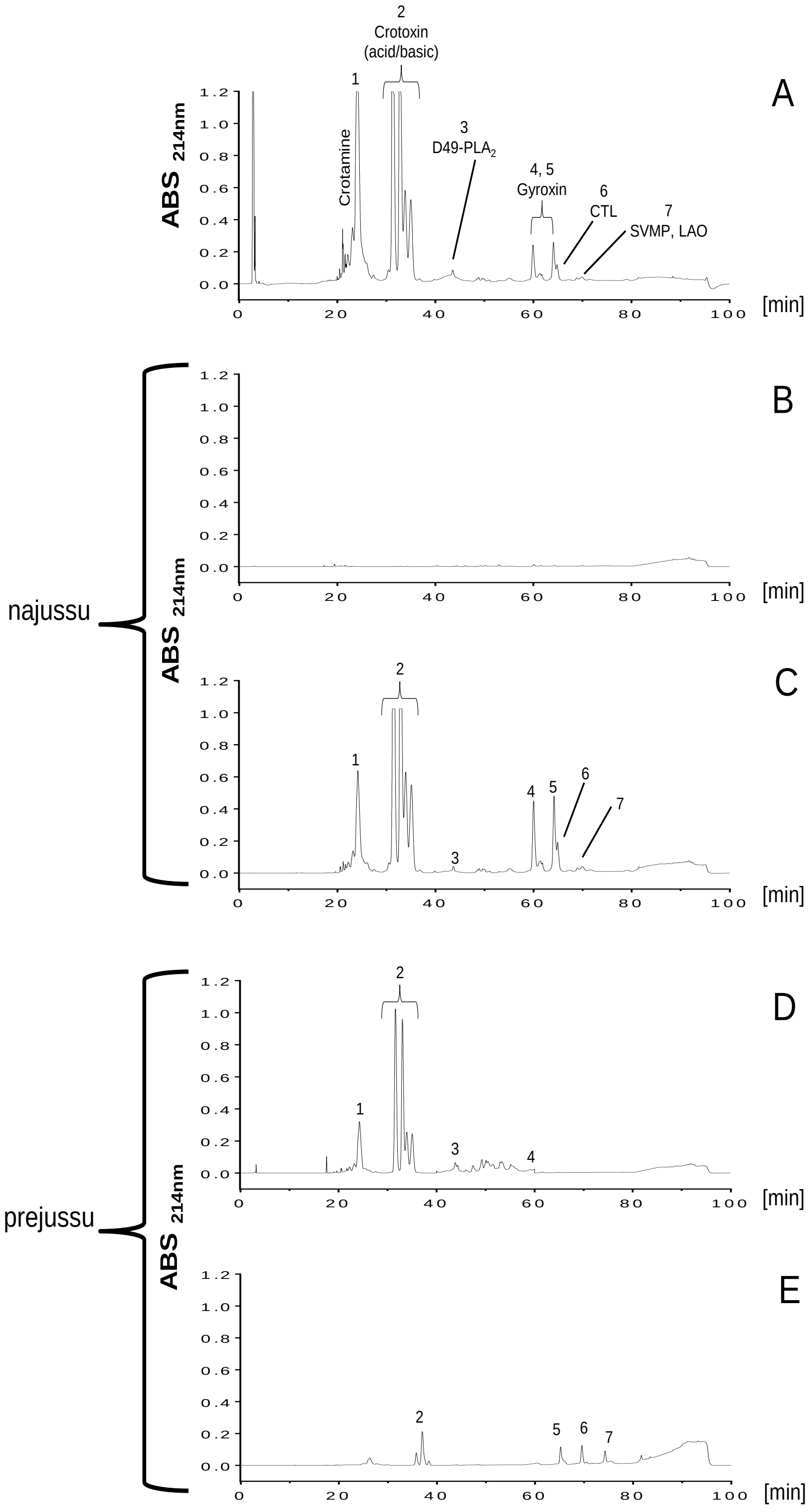

Figure S4. Antivenomic of *C. durissus* venom. Immunoreactivity was evaluated by affinity chromatography on columns containing purified najussu or prejudussu immunoglobulins. The immunocaptured and non-immunocaptured fractions of the *C. durissus* venom were analyzed by reverse phase-HPLC. In (A) crude venom profile, (B) proteins immunocaptured by najussu, (C) proteins not immunocaptured by najussu, (D) proteins immunocaptured by prejussu and in (E) proteins not immunocaptured by prejussu.
